# Piezo channels in the urinary system: Correspondence

**DOI:** 10.1038/s12276-023-01124-8

**Published:** 2023-12-01

**Authors:** Shuaishuai Hu, Xudong Yao

**Affiliations:** Department of Urology, Shanghai Tenth People’s Hospital, School of Medicine, Tongji University, Shanghai, 200072 China

**Keywords:** Bladder cancer, Diseases

Dear Editor,

We read an informative review article by Li et al.^1^, titled “Piezo channels in the urinary system”. They report that mRNA expression of Piezo2 increased significantly in both human bladder cancer tissues and mouse bladder cancer tissues. They further found that Piezo2 only correlates with tumor stage. However, this conclusion was based on the results of only one experiment. I read the original study that the author cited in the review. The study found that Piezo2 mRNA was significantly increased in the pT2 tumor stage compared to the pT0 tumor stage, but with no significant difference between the pT2 tumor stage and pT1 tumor stage. Therefore, it is not accurate to state that Piezo2 correlates with tumor stage based on this single experiment. This review provides very little information about the role of Piezo2 in bladder cancer. Here, we would like to provide more comprehensive information regarding the role of Piezo2 in bladder cancer based on current studies and tumor datasets.

There are few studies on the biological effects of Piezo2 in bladder cancer, and the conclusions are debatable. Etem et al.^2^ showed that the level of Piezo2 mRNA was significantly increased in cancerous bladder tissues compared to control bladder tissue. Regarding the Piezo2 mRNA level in different tumor stages, the study demonstrated that it was only significantly increased in the T2 tumor stage compared to the T0 tumor stage. There was no significant difference in Piezo2 mRNA level between T0 vs. T1 or T1 vs. T2 tumor stages, which suggests that Piezo2 mRNA does not correlate with tumor stage. However, in a thorough analysis of The Cancer Genome Atlas (TCGA) dataset, Liu et al.^3^ discovered that the Piezo2 mRNA level was decreased in bladder urothelial carcinoma. Furthermore, the study revealed that the gene activity of Piezo2 was higher in normal tissue than in tumor tissue. Additionally, Piezo2 mRNA expression correlated negatively with tumor mutation burden. The conflicting two studies mentioned above suggest that further experiments are needed to confirm Piezo2 mRNA or protein expression in bladder cancer and to elucidate the correlation of Piezo2 expression with tumor stages.

In conclusion, Marshall et al.^4^ identified expression of Piezo2 in lower urinary tract tissues and demonstrated its role in bladder stretch sensing and urinary function. However, there is a lack of clinical studies regarding the expression of Piezo2 in bladder cancer. Furthermore, very little is known about the pathophysiological modulation and molecular mechanisms of Piezo2 in urinary cancer. Therefore, more studies are needed to fill this knowledge gap.
